# Unique molecular signature in mucolipidosis type IV microglia

**DOI:** 10.1186/s12974-019-1672-4

**Published:** 2019-12-28

**Authors:** Antony Cougnoux, Rebecca A. Drummond, Mason Fellmeth, Fatemeh Navid, Amanda L. Collar, James Iben, Ashok B. Kulkarni, James Pickel, Raphael Schiffmann, Christopher A. Wassif, Niamh X. Cawley, Michail S. Lionakis, Forbes D. Porter

**Affiliations:** 1 Division of Translational Medicine, Eunice Kennedy Shriver National Institute of Child Health and Human Development, National Institutes of Health, DHHS, 10CRC, Rm 5-2571, 10 Center Dr, Bethesda, MD 20892 USA; 2 Fungal Pathogenesis Section, Laboratory of Clinical Immunology and Microbiology, National Institute of Allergy and Infectious Disease, National Institutes of Health, Bethesda, MD 20892 USA; 3 National Institute of Arthritis and Musculoskeletal and Skin Diseases, NIH, Bethesda, MD 20892 USA; 4 Molecular Genomics Core, Eunice Kennedy Shriver National Institute of Child Health and Human Development, National Institutes of Health, Bethesda, MD 20879 USA; 5 National Institute of Dental and Craniofacial Research, National Institutes of Health, Bethesda, MD 20879 USA; 6 National Institute of Mental Health, National Institutes of Health, Bethesda, MD 20879 USA; 7 Baylor Scott & White Research Institute, Dallas, TX USA

**Keywords:** Mucolipidosis type IV, Fabry disease, Microglia, CCL5, Neuroinflammation, Lysosomal disease

## Abstract

**Background:**

Lysosomal storage diseases (LSD) are a large family of inherited disorders characterized by abnormal endolysosomal accumulation of cellular material due to catabolic enzyme and transporter deficiencies. Depending on the affected metabolic pathway, LSD manifest with somatic or central nervous system (CNS) signs and symptoms. Neuroinflammation is a hallmark feature of LSD with CNS involvement such as mucolipidosis type IV, but not of others like Fabry disease.

**Methods:**

We investigated the properties of microglia from LSD with and without major CNS involvement in 2-month-old mucolipidosis type IV (*Mcoln1*^−/−^) and Fabry disease (*Gla*^*y*/−^) mice, respectively, by using a combination of flow cytometric, RNA sequencing, biochemical, in vitro and immunofluorescence analyses.

**Results:**

We characterized microglia activation and transcriptome from mucolipidosis type IV and Fabry disease mice to determine if impaired lysosomal function is sufficient to prime these brain-resident immune cells. Consistent with the neurological pathology observed in mucolipidosis type IV, *Mcoln1*^−/−^ microglia demonstrated an activation profile with a mixed neuroprotective/neurotoxic expression pattern similar to the one we previously observed in Niemann-Pick disease, type C1, another LSD with significant CNS involvement. In contrast, the Fabry disease microglia transcriptome revealed minimal alterations, consistent with the relative lack of CNS symptoms in this disease. The changes observed in *Mcoln1*^−/−^ microglia showed significant overlap with alterations previously reported for other common neuroinflammatory disorders including Alzheimer’s, Parkinson’s, and Huntington’s diseases. Indeed, our comparison of microglia transcriptomes from Alzheimer’s disease, amyotrophic lateral sclerosis, Niemann-Pick disease, type C1 and mucolipidosis type IV mouse models showed an enrichment in “disease-associated microglia” pattern among these diseases.

**Conclusions:**

The similarities in microglial transcriptomes and features of neuroinflammation and microglial activation in rare monogenic disorders where the primary metabolic disturbance is known may provide novel insights into the immunopathogenesis of other more common neuroinflammatory disorders.

**Trial registration:**

ClinicalTrials.gov, NCT01067742, registered on February 12, 2010

## Background

Microglia are the resident myeloid cell population of the central nervous system (CNS) and are involved in the homeostasis and immune protection of the brain [1]. They arise from progenitors in the embryonic yolk sac, migrate into the brain during development, and persist there throughout life [2]. Microglia are involved in healthy neuronal dendritic pruning during brain development and in the removal of apoptotic neurons [2, 3]. Under normal conditions, microglia maintain a highly ramified state, which can change under pathological conditions to an activated state with an amoeboid shape associated with a high phagocytic activity [3]. This switch to an activated phenotype has been observed during the progression of several neurodegenerative diseases [4–8]. Microglial activation can be triggered by signals arising from the death of either surrounding glial or neuronal cells, in response to an infection or exposure to inflammatory cytokines [1, 6].

Neuroinflammation is found in many inherited metabolic disorders and neurodegenerative diseases and has also been described in autism spectrum disorder [4, 5, 9–15]. In many of these conditions, lysosomal pathways become dysfunctional, which suggests that these pathways may be integral to the pathological neuroinflammation or neurodegeneration [11, 15, 16]. Only lysosomal storage diseases with neurodegeneration are associated with activated microglia (microgliosis) and dysregulated microglia function, which is not described in some other diseases associated with lysosomal dysfunction [5, 17, 18]. To understand the relationship between microglia activation and lysosomal dysfunction, we characterized microglia from two non-overlapping lysosomal storage diseases: Fabry disease (FD) [19] and the early stages of mucolipidosis type IV (MLIV) prior to the onset of neurodegeneration [17, 20, 21].

FD is an X-linked disorder caused by deficiency in the lysosomal enzyme α-galactosidase A [19]. Clinical manifestations in FD patients include renal failure, premature myocardial infarction, and stroke [22]. A mouse model of FD, *Gla*^*−/−*^, displays globotriaosylceramide accumulation in the lysosome; however, no significant neuropathology related to neuronal or glial cells dysfunction has been reported in these mice. In the present work, the FD mouse is used as a model for a lysosomal storage disease with minimal neurodegeneration [19]. In contrast, other lysosomal storage diseases such as MLIV, neuronopathic Gaucher and Niemann-Pick disease, type C1 and NPC1 and NPC2, and cerebroid neuronal lipofuscinosis 3 (CLN3) exhibit significant neuroinflammation and neurodegeneration [5, 23, 24].

MLIV is an autosomal recessive disorder caused by mutations in the *MCOLN1* gene encoding the calcium transporter TRPML1 [20, 21, 25, 26]. Clinical manifestations in MLIV patients include cognitive impairment, delayed motor function, and ophthalmologic abnormalities that are accurately recapitulated in the *Mcoln1*^−/−^ murine model [20, 21, 27]. This model is characterized by early gliosis that decreases with disease progression accompanied by a progressive loss of motor function in the later stages of the disease [17, 20, 28]. Although there are no current medical therapies for patients with MLIV, bone marrow transplantation and N-butyl-deoxynojirimycin (miglustat) have been shown to delay the pathologic progression in the mouse model of MLIV [28, 29].

Herein, we analyzed microglia from FD and MLIV mice and compared the mRNA expression profiles, from 2-month-old animals, with previously published microglia transcriptomes. Our results, as anticipated, show minor changes in the FD mice microglia compared to control microglia, with only the NOD-like signaling pathway altered. In contrast, microglia isolated from 2-month-old MLIV mice display an altered expression pattern similar to other neurodegenerative diseases.

## Materials and methods

### Mouse models

All mouse experiments were approved by the NICHD Animal Care and Use Committee. BALB/c-*Npc1*^+/−^ and BALB/c-*Npc2*^+/−^ mice were obtained from The Jackson Laboratory (Bar Harbor, ME). C57BL6-*Cln3*^+/−^ mice were obtained from Dr. Steven Walkley with permission provided by Dr. Susan Cotman. Fabry (C57BL6-*Gla*^y/−^) mice were provided by Dr. Ashok Kulkarni [19]; mucolipidosis type IV (C57BL6-*Mcoln1*^−*/*−^) mice were obtained from Dr. James Pickel (20); and Pompe (C57BL6-*Gaa*^−/−^) mice were obtained from Dr. Nina Raben [30]. Both the *Gla* and *Gaa* mutations were maintained as homozygotes, whereas *Cln3*, *Mcoln1*, *Npc1*, and *Npc2* were maintained as heterozygotes and intercrossed to obtain mutant and control littermates. The age of each group of animals and matched controls is specified in the corresponding figures or figure legends. Sera from 2-hydroxy-propyl-β-cyclodextrin (HPβCD)-treated animals were collected in a previous study [24].

### Human biospecimens

Serum samples were obtained from MLIV patients as part of natural history studies conducted at the NIH Clinical Center (NCT01067742). The MLIV study was approved by the NINDS Institutional Review Board. Informed consent was obtained from guardians or participants. Assent was obtained when applicable. The study was conducted in accordance with the Declaration of Helsinki.

### Microglia isolation and flow cytometry analyses

Microglia were isolated and analyzed as previously described [24, 31, 32]. Mice were euthanized using CO_2_, before cardiac perfusion with 25 mL of ice-cold PBS. Brains were removed without the spinal cord and suspended and triturated in ice-cold FACS Buffer (PBS containing 0.5% BSA (Sigma-Aldrich, St. Louis, MO, USA)) using a syringe plunger. Microglia were separated from neurons using a discontinuous Percoll gradient 30–70% (GE healthcare, Chicago, IL, USA). Microglia at the 70% interphase were collected, washed twice in FACS Buffer, and stained with fixable LIVE/DEAD fluorescent or pacific blue dye (UV-blue L-23105 or Violet L-34955, Thermo Fisher Scientific, Waltham, MA, USA)) for 10 min (1:500) in PBS at 4 °C and then incubated with rat anti-mouse CD16/32 (2.4G2; BD Biosciences, San Jose, CA, USA) for 10 min (1:100) in FACS buffer at 4 °C to block Fc receptors. For the staining of surface antigens, cells were incubated with fluorochrome-conjugated (FITC, PE, PE-Cy7, allophycocyanin [APC], APC-Cy7, APC-eFluor 780, Alexa Fluor 700, eFluor 450, eFluor 605 NC, or PerCP-Cy5.5) antibodies against mouse CD45 ((Ly-5), Biolegend, San Diego, CA, USA), F4/80 ((BM8), San Diego, CA, USA), CD11b ((M1/70), San Diego, CA, USA ), MHCII (M5/114.15.2), and CX_3_CR1 (FAB5825P, R&D System, Minneapolis, MN, USA). After three washes with FACS buffer, stained cells were fixed using 2% paraformaldehyde prior to analysis. Stained samples were analyzed using an LSR Fortessa (BD) and the data analyzed using FlowJo software (TreeStar, Ashland, OR, USA). Only single cells were analyzed; cell numbers were quantified using PE-conjugated fluorescent counting beads (Spherotech, Lake Forest, IL, USA). Additional file 1: Figure S1a shows the gating strategy used and Additional file 1: Figure S1b the indirect evaluation of cell purity based on the presence of non-microglial cell markers.

### RNA extraction and sequencing

CX_3_CR1^+^, CD45^+^, and CD11b^+^ triple-positive live cells were sorted using a FACS Aria 3 (BD) from four 2-month-old mice per condition. Cells were collected in 200 μL of FACS buffer, centrifuged at 1000×*g* for 10 min at 4 °C and the cell pellet was frozen in 700 μL TRIzol. The samples were thawed at room temperature, and 200 μL Chloroform (Sigma) was added and mixed for 15 s. The aqueous phase, after 15 min of centrifugation at 12,000×*g* at 4 °C, was mixed with anhydrous ethanol. The solution was loaded onto RNeasy micro plus columns (Qiagen, Hilden, Germany) and the total RNA was purified according to the manufacturer’s protocol. The concentration and quality of the eluted RNA was evaluated using qubit and PicoChip Bioanalyzer. RNA-Seq libraries were constructed using SMARTer Stranded Total RNA-Seq Kit - Pico Input Mammalian (Takara Bio USA, Inc. Mountain View, USA). In brief, 2 ng of total RNA from each sample was fragmented and reverse-transcribed to cDNA, and then adapters for Illumina sequencing (Illumina, San Diego, CA, USA) (with specific barcodes) were added through PCR. The PCR products were purified, and then ribosomal cDNA was depleted using probes specific to mammalian rRNA. The cDNA fragments were further amplified with primers universal to all libraries. These libraries were pooled together and sequenced using the Illumina HiSeq 2500 to generate approximately 40 million 2 × 100 paired-end reads for each sample. The raw data was demultiplexed and analyzed further. Sequence alignment was performed using RNA-STAR [33], quantitation was performed with subread featureCounts [34], and comparisons of expression was performed with DESeq2 [35]. The raw data is available under the GEO accession number GSE112895.

### Measurement of reactive oxidative stress

2′,7′-Dichlorofluorescin diacetate (DCFDA; Sigma-Aldrich, St. Louis, MO, USA) staining was performed according to the manufacturer’s instructions. In brief, 10,000 FACS-sorted microglia per well were seeded just after isolation and allowed to attach for 2 h to the bottom of fluorescence 96 well plates (Sigma-Aldrich, St. Louis, MO, USA). The wells were washed twice with 100 μL PBS and stained for 30 min with 20 μM DCFDA solution per well before reading with an automated plate reader with Ex/Em 488/520 nm (BMG Labtech, Ortenberg, Germany).

### Measurement of energy metabolism

Real-time measurements of extracellular acidification rates, a measure of lactate production, were performed on an XF96e Seahorse extracellular flux analyzer (Agilent Technology, Santa Clara, CA, USA) according to the manufacturer’s instructions. Measurement was performed on 10,000 FACS-sorted microglia.

### ELISA and western blots

Total protein was extracted from FACS-sorted microglia using radioimmunoprecipitation assay (RIPA) buffer (50 mM Tris–HCl, pH 7.4, 1% NP-40, 0.5% sodium deoxycholate, 0.1% SDS, 150 mM NaCl, 2 mM EDTA, 40 mM NaF, 0.2 mM Na_3_VO_4_) with complete protease inhibitor cocktail (Roche, Basel, Switzerland). Protein amounts were determined using the DC assay (Bio-Rad Laboratories, Hercules, USA). Protein lysates (typically 5–20 μg) were loaded onto a 4–12% gradient NuPAGE Gel, and electrophoresis was carried out at a constant 120 V. Protein transfer was performed using the iBLOT2 dry transfer setup (25 V, 12 min). Following transfer, nitrocellulose membranes were incubated in a 2% BSA and PBS 0.1% Tween 20 blocking buffer for 1 h at room temperature, followed by incubation with the primary antibody at 4 °C overnight. After the initial incubation, the membrane was rinsed and incubated with the appropriate secondary antibody for 1 h at room temperature. Membrane development was carried out using the Bio-Rad Chemiluminescence Detection Kit (Bio-Rad Laboratories). Primary antibodies used include ERK1/2 (1/1000, Cell Signaling Technology (CST), Denver, USA), Phospho-ERK1/2 (1/1000, CST), HIF1α (1/800, Sigma), iNOS (1/800, Sigma), β-tubulin (1/2000, Sigma), anti-mouse HRP, anti-goat HRP or anti-rabbit HRP (1/20,000, Sigma), CTSD (1/500, Santa Cruz Biotechnology, USA), GAPDH (1/2000, CST), DAP12 (1/1000, Thermo Fisher Scientific, Waltham, USA), and APOE (1/500, Biodesign, Saco, USA). ELISA anti-CCL5 human or mouse was performed according to the manufacturer’s instructions (Thermo Fisher Scientific). VTS-270-treated mouse sample was collected in a previous study [24].

### Immunofluorescence

Mice were euthanized by CO_2_ asphyxiation and transcardially perfused with PBS followed by ice-cold 4% paraformaldehyde in PBS, pH 7.4. The brains were postfixed in 4% paraformaldehyde solution for 24 h and then cryoprotected in 30% sucrose (Sigma-Aldrich, St. Louis, MO, USA). Cerebellar tissues were cryostat-sectioned parasagittally (20 μm) and floating sections were collected in PBS with 0.25% Triton X-100 (Sigma-Aldrich, St. Louis, MO, USA). Sections were incubated overnight at 4 °C with rabbit anti-IBA1 (1/200, Wako, Richmond, VA, USA), rat anti-CD68-PE (1/100, FA-11, Biolegend, San Diego, CA, USA), and chicken anti-GFAP (1/500, nbp1-05198, Novus, Centennial, CO, USA). Secondary antibodies prepared in goat and conjugated with Alexa-488, Alexa-594, or Alexa-647 and Hoechst3342 (1/1000 and 1/5000, respectively; Thermo Fisher Scientific, Waltham, MA, USA) were incubated for 1 h at room temperature. Images were taken using a Zeiss Axio Observer Z1 microscope fitted with an automated scanning stage, Colibri II LED illumination and Zeiss ZEN2 software using a high-res AxioCam MRm camera and a 20× objective. Each fluorophore channel was pseudo-colored in ZEN2, exported as CZI, and analyzed using ImageJ [36].

### Datasets, pathway, and statistical analysis

Published datasets used for comparison in our study include those from *App*, *Sod1*^*G93A*^, *Ercc1*, and aged mice [15] and Niemann-Pick type C1 [24]. Results are presented as mean ± SD unless otherwise specified. A Mann-Whitney *U* test was used to compare differences between groups. Differences were considered significant if the *p* value was *p < 0.05, **p < 0.01, and ***p < 0.001. Statistical calculations were performed using GraphPad Prism software version 5 (San Diego, USA). Heatmap and clustering were performed with Morpheus https://software.broadinstitute.org/morpheus/. Pathway analysis was performed using WebGSALT [37], g:Profiler [38], STRING [39], and ConsensusPathDB [40]. Default setting was used for all four web resources. A significant enrichment for a pathway was set at *p* < 0.05 and the fold difference cutoff was set at twofold change for up- and downregulated genes.

## Results

### Microglia phenotypes are lysosomal storage disease-specific and mirror the presence of corresponding neurological manifestations

**Fig. 1:**
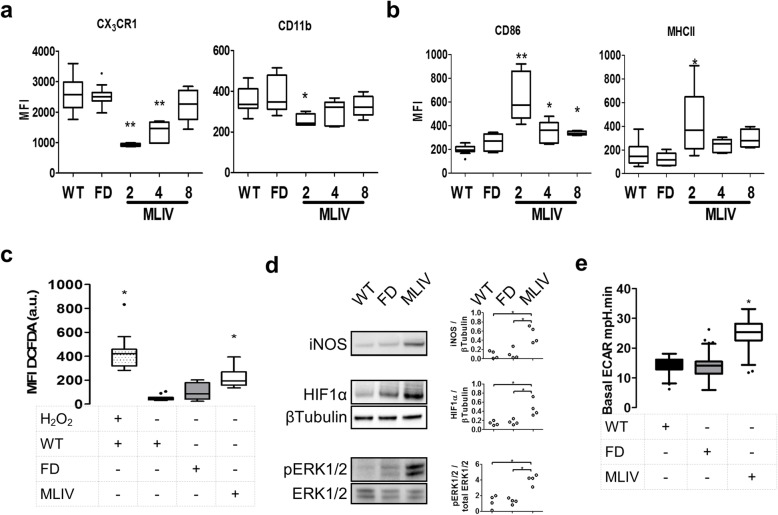
Analysis of the microglia in Fabry disease and mucolipidosis type IV mice. Mean fluorescence intensity (MFI) of the surface expression of microglial lineage markers **a** CX3CR1 and CD11b and microglial activation markers. *N* ≥ 5; *p < 0.05, **p < 0.01 Mann-Whitney *U* test vs. WT. **b** CD86 and MHCII. WT, control; FD, 2-month-old Fabry disease; MLIV, 2-, 4-, and 8-month-old mucolipidosis type IV. *N* ≥ 5; *p < 0.05, **p < 0.01 Mann-Whitney *U* test vs. WT. **c** DCFDA analysis of free radical production by 10,000 microglial cells. 1 μM H_2_O_2_ treatment was used as a positive control for free radical production. *N* = 5, *n* ≥ 3; *p < 0.05, **p < 0.01 Mann-Whitney *U* test vs. WT. **d** Western blot analysis iNOS, P-ERK1/2, ERK1/2, and HIF1α and β-tubulin on 100,000 microglia lysates. *N* = 4, *p < 0.05 Mann-Whitney *U* test. **e** Basal extracellular acidification rate (ECAR) in mpH.minutes^−1^ of unbuffered seahorse media by 10,000 cells. *N* = 5, *n* ≥ 3; *p < 0.05 Mann-Whitney *U* test vs. WT

*Mcoln1*^−/−^ mice exhibit early gliosis, which decreases over time without reaching control levels [17], unlike the aged-matched *Gla*^y/−^ mice that do not present with gliosis and do not exhibit neurological deficits at 2 months of age [24]. We previously showed that *Mcoln1*^−/−^ microglia had normal granularity and size at 4 months of age [24]. However, analysis of microglia and myeloid lineage markers (CX_3_CR1, CD11b (Fig. 1a)) and activation markers (CD86, MHCII (Fig. 1b)) showed significant changes in *Mcoln1*^−/−^ (MLIV) when compared to either littermate controls (WT) or *Gla*^y/−^ (FD) mice. Although with a decreasing trend between 2 and 8 months of age, CD86 surface expression was significantly elevated relative to control mice at all ages (Fig. 1a), supporting prior observations [17]. A time course analysis of CX_3_CR1 and CD11b surface expression showed significantly decreased levels at 2 months of age, with increases toward control levels between 2 and 8 months of age (Fig. 1a). Analysis of MHCII expression showed significantly increased expression only in 2-month-old *Mcoln1*^−/−^ animals with normalization thereafter. Analogous investigations, performed on brains from other LSD mouse models (Gaucher [41], Sandhoff [42], NPC1 [13], and NPC2 and CLN3 (unpublished data)) also displayed an altered morphology and surface marker expression levels on their microglia.

We previously reported increased free radicals and a switch to glycolytic metabolism in activated NPC1 microglia [24]. Similar to what we observed in NPC1 mice, the free radical content was significantly increased in microglia isolated from 2-month-old MLIV mice (Fig. 1c). As expected, based on the absent microglial activation observed in FD mice, the free radical content was not increased in *Gla*^y/−^ microglia (Fig. 1c). Consistent with these results, we also observed increased expression of iNOS in MLIV, but not FD, microglia (Fig. 1d). Glycolytic metabolism was evaluated by measuring extracellular medium acidification rate (ECAR), which was significantly increased in microglia obtained from MLIV mice, but not FD microglia, indicative of a shift to glycolytic metabolism (Fig. 1e). The metabolic shift measured by the increased ECAR in MLIV microglia was supported by the observation of increased expression of HIF1α and pERK1/2 (Fig. 1d), two key effector pathways in the regulation of energy metabolism [43].

### Microglia transcriptomic changes are lysosomal storage diseases specific

**Fig. 2:**
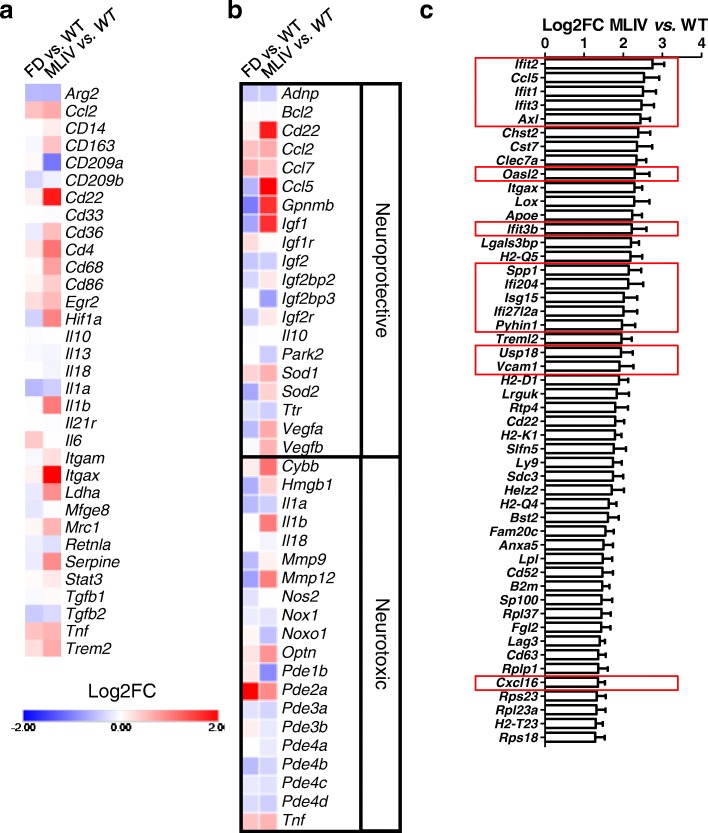
Microglia transcriptome analysis. **a** Heatmap of the microglia/macrophage activation markers in MLIV and FD expressed as Log2 fold change relative to control levels. **b** Heatmap of neurotoxic and neuroprotective markers in MLIV and FD expressed as Log2 fold change relative to control levels. The genes in this list are based upon [16, 24, 44]. **c** Top 50 most differentially overexpressed genes in MLIV compared to control microglia. Interferon signaling genes are boxed in red. Data underlying the heatmaps are provided in Additional file 5: Table S1 and Additional file 6: Table S2

In order to better understand the phenotypic changes observed in MLIV microglia, we performed RNA sequencing of microglia isolated from *Mcoln1*^−/−^ brain. The transcriptomic profile was then compared to the transcriptomes of microglia isolated from wild-type and FD mouse brain. Transcriptome analysis of *Gla*^y/−^ microglia identified few genes with significantly differential expression relative to age-matched wild-type control microglia (i.e., 44 upregulated and 42 downregulated genes (Additional file 5: Table S1, Additional file 1: Figure S1)). The small number of differentially expressed genes between wild-type and *Gla*^y/−^ microglia is in support of the absent gliosis and lack of a neurodegenerative phenotype in 2-month-old FD mice. Pathway analysis using four different software programs consistently identified the upregulation of only the NOD-like receptor signaling pathway in *Gla*^y/−^ microglia compared to aged-matched wild-type control microglia (Additional file 1: Figure S1). In addition, in agreement with the lack of significant neuroinflammation in *Gla*^y/−^ mice, analysis of common markers of neuroinflammation and microglial activation revealed only minor changes (Fig. 2a).

In contrast to *Gla*^y/−^ microglia, transcriptomic analysis of microglia isolated from MLIV animals showed major expression differences compared to microglia from littermate wild-type control animals. Specifically, we found 386 significantly upregulated and 44 significantly downregulated genes in MLIV relative to wild-type microglia (Additional file 6: Table S2, Additional file 2: Figure S2). Pathway analysis performed on these differentially expressed genes demonstrated changes in the expression of genes related to lysosomal function, activation of immune cells (i.e., complement, antigen presentation and processing, and rheumatoid arthritis) and neurodegeneration (Alzheimer’s, Parkinson’s, and Huntington’s diseases, Additional file 2: Figure S2). The RNA expression data (Fig. 2a) are indicative of microglial activation, in support of our phenotypic analyses that showed increased expression of CD86 (Fig. 1b) and HIF1α (Fig. 1d). In addition, consonant with the increased expression of *Hif1a*/HIF1α and the functional shift to glycolytic metabolism, we observed significantly increased expression of the known HIF1 target genes *Aldoa*, *Bnip3*, *Egln3*, *Igf1*, *Nampt1*, *Pgk1*, and *Vegfa* (Additional file 6: Table S2) [45].

The RNA expression profile in MLIV microglia showed a mixed neuroprotective/neurotoxic pattern (Fig. 2b), similar to what was previously observed in NPC1 microglia, amyotrophic lateral sclerosis (ALS), and glioblastoma [16, 24, 44]. Specifically, there was a significant increase in expression of neurotoxic-related genes such as *Cd22*, *Il1b*, and *Cybb*, while at the same time, there was a significant increase in expression of genes generally regarded as neuroprotective such as *Ccl5*, *Igf1*, and *Gpnmb* (Fig. 2b). The expression of genes associated with both neurotoxic and neuroprotective function suggests a complex role played by microglia in MLIV. Of note, advances in single-cell transcriptomic analyses have shown that in both ALS and Alzheimer’s disease (AD) (SOD1^G93A^ and 5xFAD models, respectively), a continuum of ramified to amoeboid microglial cells is present in the mouse brain with “disease-associated microglia” (DAM) present in the brain areas affected by pathology [46]. Indeed, we found the DAM marker *Cd11c*/*Itgax* to be significantly overexpressed in MLIV microglia (Fig. 2a, Additional file 6: Table S2)

Of the 50 most significantly upregulated genes in MLIV microglia, 15 (30%) are related to interferon signaling (Fig. 2c). From our in silico analysis, most of these genes are regulated by five different interferon regulatory factors: IRF1 (9 out of 50), IRF2 (5 out of 50), IRF3 (7 out of 50), IRF7 (7 out of 50), and IRF9 (10 out of 50). It has been suggested that the transcription factor EB (TFEB) could potentially be important in MLIV pathology [47]; however, it does not appear to regulate the 50 most significantly overexpressed genes in *Mcoln1*^−/−^ microglia in our analysis.

### Elevated CCL5 in microglia and serum of MLIV mice

**Fig. 3:**
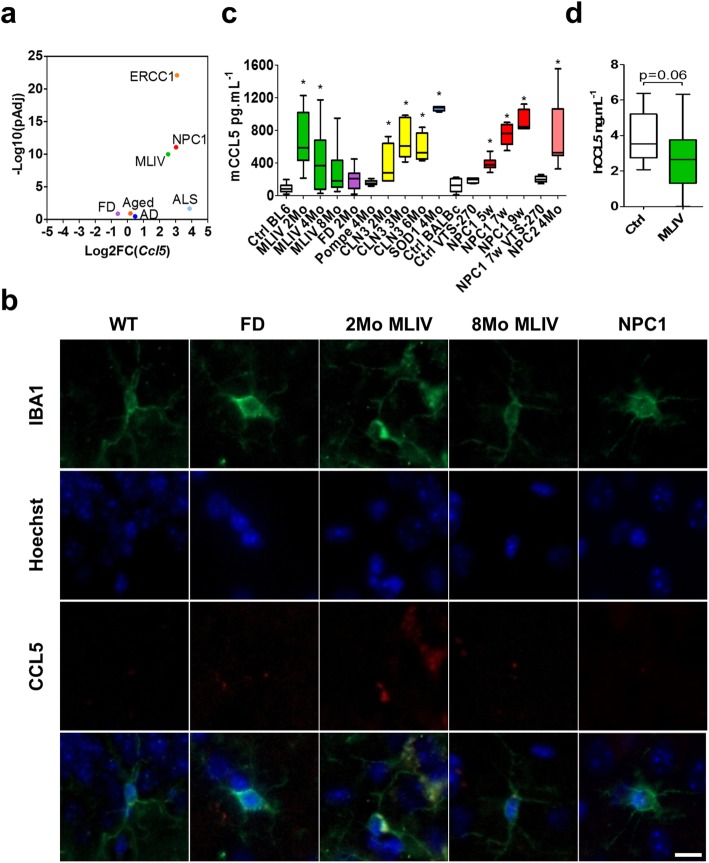
Analysis of CCL5 expression in microglia. **a** *Ccl5* Log2 fold change and Log10 (pAdj) in 2-month-old Fabry disease (FD), 7-week-old Niemann-Pick type C1 (NPC1), 3-month-old amyotrophy lateral sclerosis (SOD1), 16-month-old App-Ps1 (AD), and 2-month-old mucolipidsosis type IV (MLIV) microglia isolated from mouse brain **b** IBA1 and CCL5 double staining in hippocampi. The scale bar is 10 μm. **c** CCL5 quantification by enzyme-linked immunosorbent assay (ELISA) in mouse serum. *Npc1*^*−/−*^ *N* = 7, MLIV *N* = 8, FD *N* = 16, 2-month-old *Gaa*^−/−^ (Pompe disease) *N* = 10, 3-month-old *Npc2*^−*/*−^ *N* = 7, *Sod1*^*tg*G93A^ *N* = 4, 2-, 3-, and 6-month-old *Cln3*^−*/*−^ *N* = 4–6. HPβCD/VTS-270-treated animals were subcutaneously injected every other day from day 7 to 49 [24], *N* = 4 and 5 for control and *Npc1*^−*/*−^, respectively. **d** CCL5 ELISA from control and MLIV patient’s serum. *N*_control_ = 14, *N*_MLIV_ = 48

We observed the chemokine *Ccl5* (RANTES) to be highly overexpressed, second only to *Ifit2*, in microglia isolated from MLIV mice (Fig. 2c). Increased expression of *Ccl5* has been reported in other mouse models with neurodegeneration and neuroinflammation including Gaucher [23], NPC1 [23], Sandhoff [23], Krabbe [23], AD [15, 48], and ALS [16], as well as during aging [15] (Fig. 3a). Whole brain immunofluorescence staining showed strong CCL5 signal in the hippocampus, but not other brain areas, of 2-month-old MLIV mice. Co-immunostaining with the microglial marker IBA1 indicated CCL5 expression within microglia (Fig. 3b, Additional file 2: Figure S2). Micsenyi et al. previously reported pathological defects in the hippocampus of MLIV mice [20]. In *Npc1* mutant mice, CCL5 immunostaining was observed in the cerebellar tissue (Additional file 3: Figure S3), a brain region extensively documented to have neuronal loss and microgliosis. The brain region-specific expression of CCL5 within microglia suggests that CCL5 may be a functional role in the pathological process. Given that CCL5 is a secreted protein, we measured its level in the serum of MLIV mice and observed a significant increase at 2 and 4 months, but not at 8 months, when compared to levels found in wild-type control mice (Fig. 3c). This observation is in agreement with the decrease in gliosis occurring in the MLIV mouse brain between 4 and 8 months (Fig. 1b) [17]. Given that *Ccl5* expression is increased in other disorders of neuroinflammation [23, 41, 49, 50], we measured serum CCL5 levels in other mouse models of lysosomal storage and neurodegenerative diseases. Significant increases in CCL5 serum levels were observed in ALS, CLN3, NPC1, and NPC2 compared to wild-type control mice (Fig. 3b). The progressive increase in CCL5 in NPC1 and CLN3 with disease progression, which mirrors the MLIV pattern, supports the previously reported increase inflammation over time in these pathologies [5, 24, 51]. Consistent with the lack of neuroinflammation, CCL5 serum levels were not increased in either Pompe or FD mice (Fig. 3c). To determine if reducing neuroinflammation correlated with a change in sera concentration of CCL5, we measured CCL5 levels in the serum of NPC1 mice subcutaneously injected with the most potent therapeutic compound in this disease model: 2-hydroxy-propyl-β-cyclodextrin (HPβCD/VTS-270) at 4 g/kg [24], and observed a significant decrease toward normal (Fig. 3c).

Given the temporally related changes in serum CCL5 levels observed in NPC1, CLN3, and MLIV mutant mice, we wondered whether serum CCL5 levels could be used as a biomarker in MLIV patients. A decreased CCL5 concentration in the serum of 48 MLIV patients compared to control individuals (Fig. 3d) did not support the animal results. Nonetheless, with respect to its utility as a biomarker, serum CCL5 levels did not appear to correlate with either patient age or disease severity [52].

### Comparison of microglia transcriptomes from mice with different neuroinflammatory syndromes

**Fig. 4:**
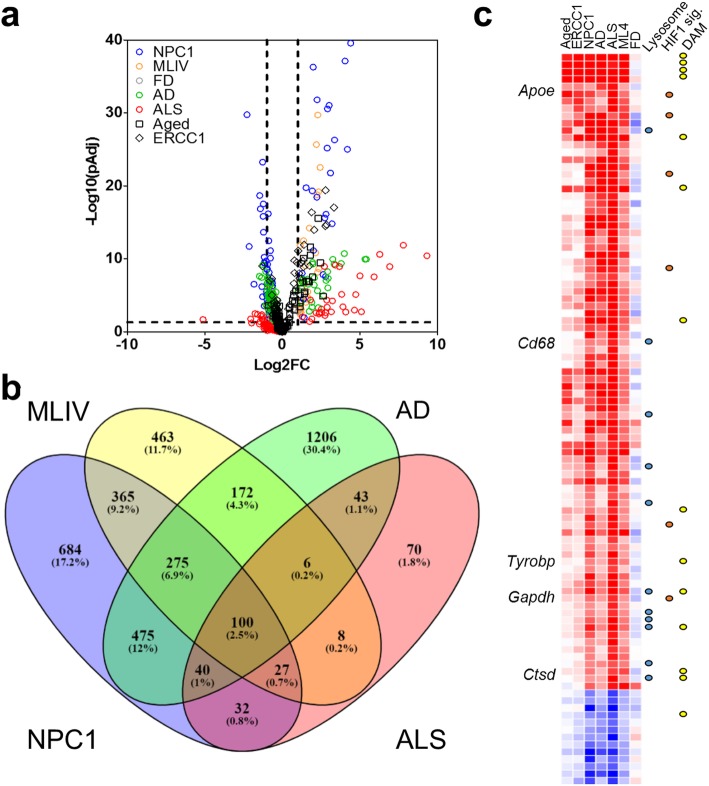
Comparison of microglia transcriptomes in diseases with neuroinflammation. **a** Volcano plot differentially expressed genes *X* = Log_2_ fold change vs control, *Y* = − Log_10_(*p*). The data are supplied in Additional file 7: Table S3. The dashed lines are set at the *p*Adj of 0.05 and Log2 fold change of – 1 and 1 on *Y*- and *X*-axis, respectively. **b** Venn diagram of the significantly differentially expressed genes (*p*Adj < 0.05, Log2FC > 1) genes in ALS (SOD1), AD (APP), NPC1, and MLIV disease mouse models. **c** Heatmap of the 100 modified genes AD, ALS, MLIV, and NPC1 diseases across aging (aged and ERCC1) and FD datasets. Yellow: DAM markers. Orange: HIF1 signaling. Blue: lysosome. The heatmap with all genes labeled is provided in Additional file 4: Figure S4a

Pathway analysis of differentially expressed genes in MLIV microglia showed an overlap with other neurodegenerative disorders including Parkinson’s, Alzheimer’s, and Huntington’s diseases (Additional file 2: Figure S2). To further investigate this observation, we extracted microglia transcriptomes from Gene Expression Omnibus [53]. Among the extracted transcriptomes, five are from mouse models known to have significant neuroinflammation: these include 18-month-old and *Ercc1*^*Δ/KO*^ mice [54], which represent aging models, and three from NPC1, AD (amyloid precursor protein model), and ALS (SOD1^G93A^) diseases (Fig. 4a). Analysis of the significantly modified genes found in NPC1, AD, and ALS transcriptomes together with the MLIV microglia transcriptome revealed 100 genes that were significantly modified and followed the same expression pattern in all four disease models (Fig. 4b, c; Additional file 4: Figure S4); 13 genes were downregulated and 87 were upregulated among these 100 genes. Notably, 14 of the overexpressed genes (Fig. 4c, Additional file 4: Figure S4**)** have been associated with a DAM signature of microglia, previously described in both the 5xFAD mouse model of AD and the SOD1^G93A^ mouse model of ALS [46]. Hence, independent of the underlying pathological condition, these genes appear to follow the same pattern with microglia from the two aging models (Fig. 4b) and none of these genes exhibited the DAM pattern in microglia isolated from FD mice. To validate this observation, we evaluated the protein expression level of APOE, GAPDH, CTSD, CD68, and DAP12. In our experiment, we were not able to measure the statistical difference in the expression level of GAPDH between the different groups. Significantly increased expression of HIF1a (Fig. 1), DAP12 (Additional file 4: Figure S4b), and APOE (Additional file 4: Figure S4b) was measured by western blot when comparing ML4 to WT (*p* < 0.05, *N* = 4, Mann-Whitney test). CD68 and CTSD expression levels were evaluated by double immunofluorescence with IBA1 (Additional file 4: Figure S4c), and positive double staining was observed in the hippocampus of 2-month-old ML4 mice.

## Discussion

Neuroinflammation is a common feature shared by several neurodegenerative diseases and a subset of rare LSD [16, 55]. Most of the data available have focused on the neurotoxicity or protective function of the microglia in the context of the studied pathology. Transcriptomic analysis of whole brain-isolated microglia revealed no major differences between healthy and non-reactive microglia in the context of FD. A similar lack of activation of the microglia also occurs in Pompe disease, a lysosomal glycogen storage disease (unpublished observation). The absence of activation is supported by functional analysis of microglia metabolism and free radical production which are unmodified.

One limitation of the current manuscript is that we have not established that the pattern of microglia activation observed in the MLIV mouse model is applicable to the human disorder. Future studies could explore expression of DAM markers in human brain tissue. In addition, in the present work, we report *Ccl5*/CCL5 as one of the most significantly overexpressed gene in mouse models of lysosomal storage diseases with neuroinflammation correlating with the microgliosis. This observation is not restricted to lysosomal storage diseases but did not translate to human, limiting its potential as a biomarker in MLIV. The use of CCL5 as a biomarker in neurodegenerative diseases has been controversial with conflicting results depending on the study [49, 56].

A similar upregulation of genes in IFN pathway has been observed in microglia from neuronopathic Gaucher and Krabbe disease mice [23], but not in NPC1 or Sandhoff disease animals [23]. Thus, activation of interferon signaling may only occur in a specific subset of LSD. However, Vitner et al. [23] showed that genetic ablation of *Ifnar1*, a key effector receptor of type I interferon signaling, in Gaucher mice had no impact on survival. Therefore, it is unclear how interferon signaling is involved in the pathological cascade of these LSD, including in MLIV; future studies examining genetic or pharmacological ablation of type I interferon signaling in MLIV mice will be required to shed light into this important question.

Functional and transcriptomic analysis supports the premise that microglia activation is driven by surrounding cells, “death,” rather than the sole loss of lysosomal protein or their functions. Comparison of transcriptomes shows similarities between neurodegenerative disease, AD, aging, ALS, MLIV, and NPC1. Based on this observation, treatments reducing microglial activation in the first three diseases and reducing disease burden should be evaluated in MLIV. Work by Boudewyn et al. [29] have shown that miglustat, a drug which decreases microgliosis in NPC1 mice, is effective in MLIV. Based on these observations, we could expect the inhibition of receptor-interacting protein kinase 1 or 3, which have been suggested to reduce the neuroinflammation in lysosomal storage diseases [57, 58] and could be considered for therapeutic intervention in MLIV.

Among the 87 genes with increased expression in the four disorders with neuroinflammation, 11 encode for lysosomal proteins, indicating that lysosomes may be involved in microglial dysregulation in neurodegenerative diseases and, to a lesser extent, in aging. The last enriched gene set includes HIF1-responsive genes (5/87), a pathway involved in microglia activation and energy metabolism [59]. This pathway or its target genes have previously been shown to be overexpressed in microglia from AD, ALS, and NPC1 mice [16, 24, 60].

The microglial transcriptomic signature analysis of disease state suggests a strong overlap between disease and aging. A subset (14%) of the shared modified genes in neurodegenerative disease and aging is DAM-associated markers, suggesting these cells might be participating in the pathology of MLIV. Our results cannot define if the presence of microglia in the pathologic areas is beneficial or deleterious; however, they strengthen the relevance of studying these specific microglia subpopulations. The recent demonstration of anti-CD22 treatment effectiveness to improve memory function in aged mice [61] suggests this approach could be applied to MLIV which like NPC1 and aged animals display a significant overexpression of this gene.

## Conclusions

The results presented in this paper for MLIV microglia, along with our prior characterization of NPC1 microglia, suggest that microglia from common and rare neurodegenerative diseases share similarities in their activation pattern. Specifically, transcriptome and functional analysis indicate that both NPC1 and MLIV microglia resemble microglia isolated from AD and ALS mouse models. Our results thus strengthen the concept of a disease-associated microglia population in these disorders with neuroinflammation. Based on these observed homologies, therapeutic approaches modulating microglia function may be applicable to both common and rare disorders.

## Supplementary information

**Additional file 1: Figure S1**. Gating strategy and cell purity evaluation. a, Representative FACS plot of 2-month-old wild type, FD and ML4 (from left to right) microglia. **b**, Bar graph of the Read counts in each replicate for monocyte/macrophage specific (*Pf4*), oligodendrocyte (*Mog* and *Sox10*), Neuron (*Map2*, *Snap25*, *Isl2* and *Mnx2*), astrocyte (*Aldh1a1* and *S100b1*) and microglia (*Cx3cr1*, *Tmem119* and *P2ry12*) markers.

**Additional file 2: Figure S2**. Transcriptomic analysis of 2-month-old Fabry Disease and Mucolipidosis type IV mice microglia. Differential expression was set at + or – 1Log2-fold divergence *vs*. control with pAdj<0.05. *p<0.01, **p<0.001. Pathway analysis performed with gProfiler (A), ConsensusPathDB (B), STRING (C) and WebGSALT default settings. Data are in Additional file 5: Table S1 and Additional file 6: Table S2.

**Additional file 3: Figure S3**. Double immunostaining for IBA1 and CCL5 in cerebellar section from 2-month-old wild type (WT), FD, MLIV and NPC1 mice. The scale bar is 20 μm.

**Additional file 4: Figure S4**. Expanded Fig. **4****c. a** Enlarged heatmap with all genes labeled. **b** Western blot analysis of GAPDH, B-Actin, DAP12, APOE on 100,000 microglia lysates. N=4. **c** representative IBA1/CD68 immunostaining of WT, FD and ML4 mice hippocampus (DG). **d** representative IBA1/CTSD immunostaining of WT, FD and ML4 mice hippocampus (DG). Scale bare is 10 μm.

**Additional file 5: Table S1**. List of genes expressed in Fabry disease mice microglia with expression as log2 fold change *vs.* WT.

**Additional file 6: Table S2**. List of genes expressed in mucolipidosis type IV mice microglia with expression as log2 fold change *vs.* WT.

**Additional file 7: Table S3**. raw data used to build Fig. **4****a** with expression as log2 fold change *vs.* controls.

## Data Availability

The sequencing data are available on Gene Expression Omnibus under the accession number GSE112895. Mouse models are available from the Jackson Laboratory.

## Abbreviations

AD: Alzheimer’s disease
ALS: Amyotrophic lateral sclerosis
CLN3: Neuronal ceroid lipofuscinosis 3
DAM: Disease-associated microglia
DCFDA: 2′,7′-Dichlorofluorescin diacetate
FD: Fabry disease
HIF1: Hypoxia inducible factor 1
IRF: Interferon regulatory factor
MLIV: Mucolipidosis type IV
WT: Wild type
