# Supplementary figures and images for: Unique molecular signature in mucolipidosis type IV microglia
Source: J Neuroinflammation. 2019 Dec 28;16:276. doi: 10.1186/s12974-019-1672-4 (PMC6935239; doi:10.1186/s12974-019-1672-4)

Figure S1

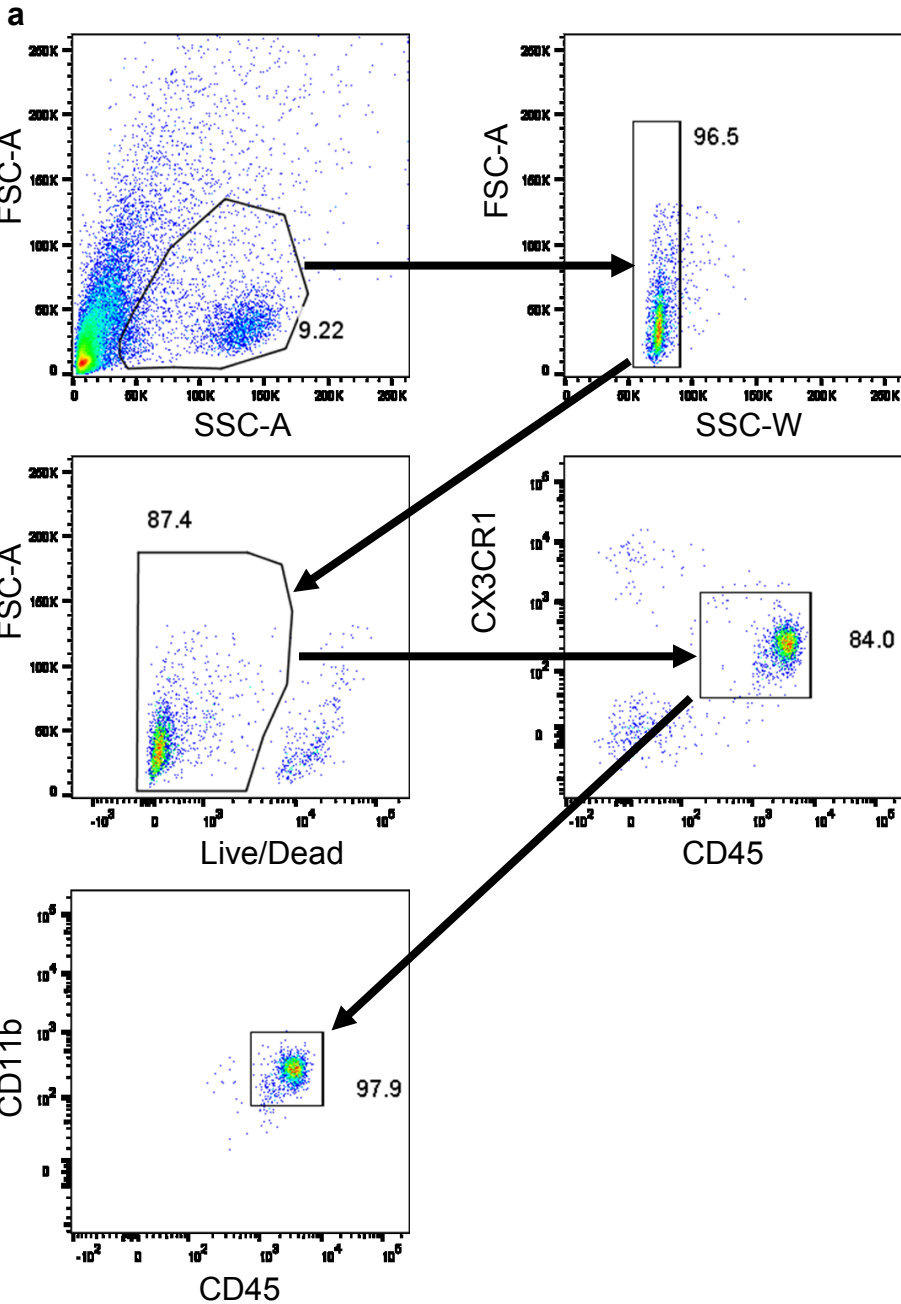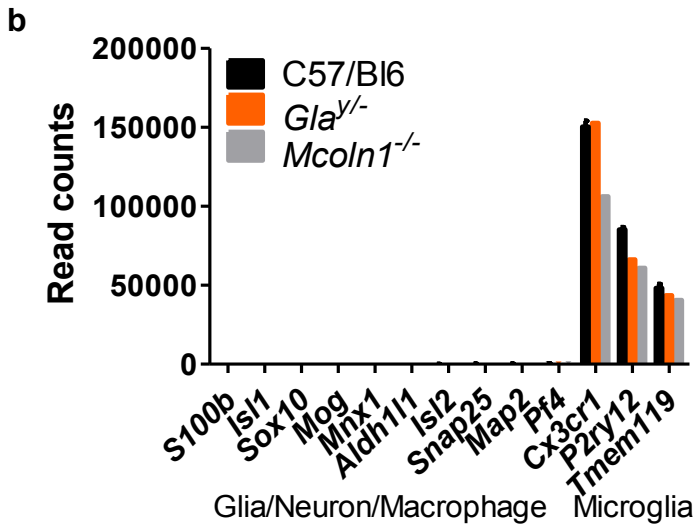

Supplement: Supplementary file 1 — Additional file 1: Figure S1. Gating strategy and cell purity evaluation. a, Representative FACS plot of 2-month-old wild type, FD and ML4 (from left to right) microglia. b, Bar graph of the Read counts in each replicate for monocyte/macrophage specific (Pf4), oligodendrocyte (Mog and Sox10), Neuron (Map2, Snap25, Isl2 and Mnx2), astrocyte (Aldh1a1 and S100b1) and microglia (Cx3cr1, Tmem119 and P2ry12) markers. [file 12974_2019_1672_MOESM1_ESM.pdf]

Fig. S3

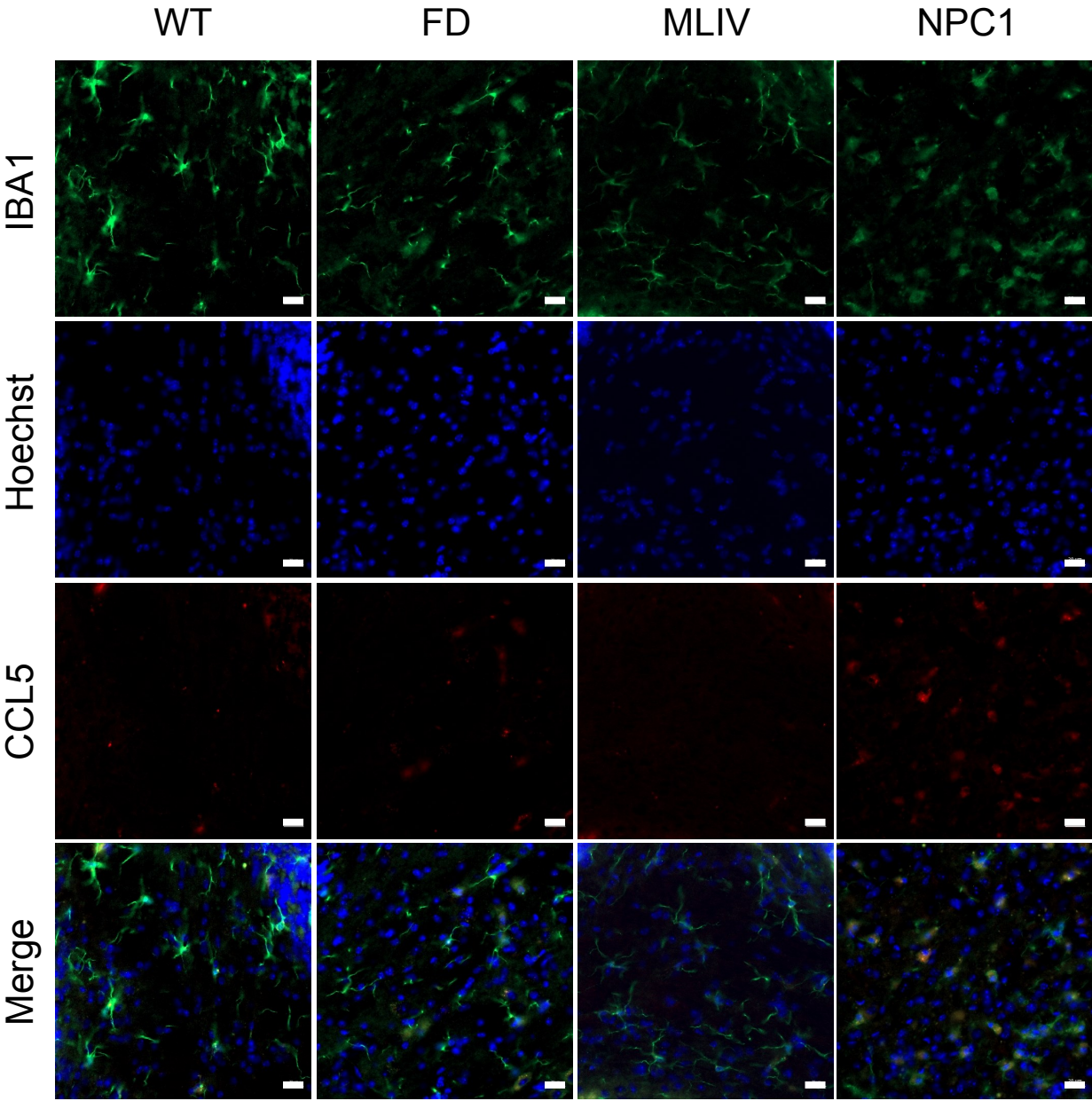

Supplement: Supplementary file 3 — Additional file 3: Figure S3. Double immunostaining for IBA1 and CCL5 in cerebellar section from 2-month-old wild type (WT), FD, MLIV and NPC1 mice. The scale bar is 20 μm. [file 12974_2019_1672_MOESM3_ESM.pdf]

Fig. S4

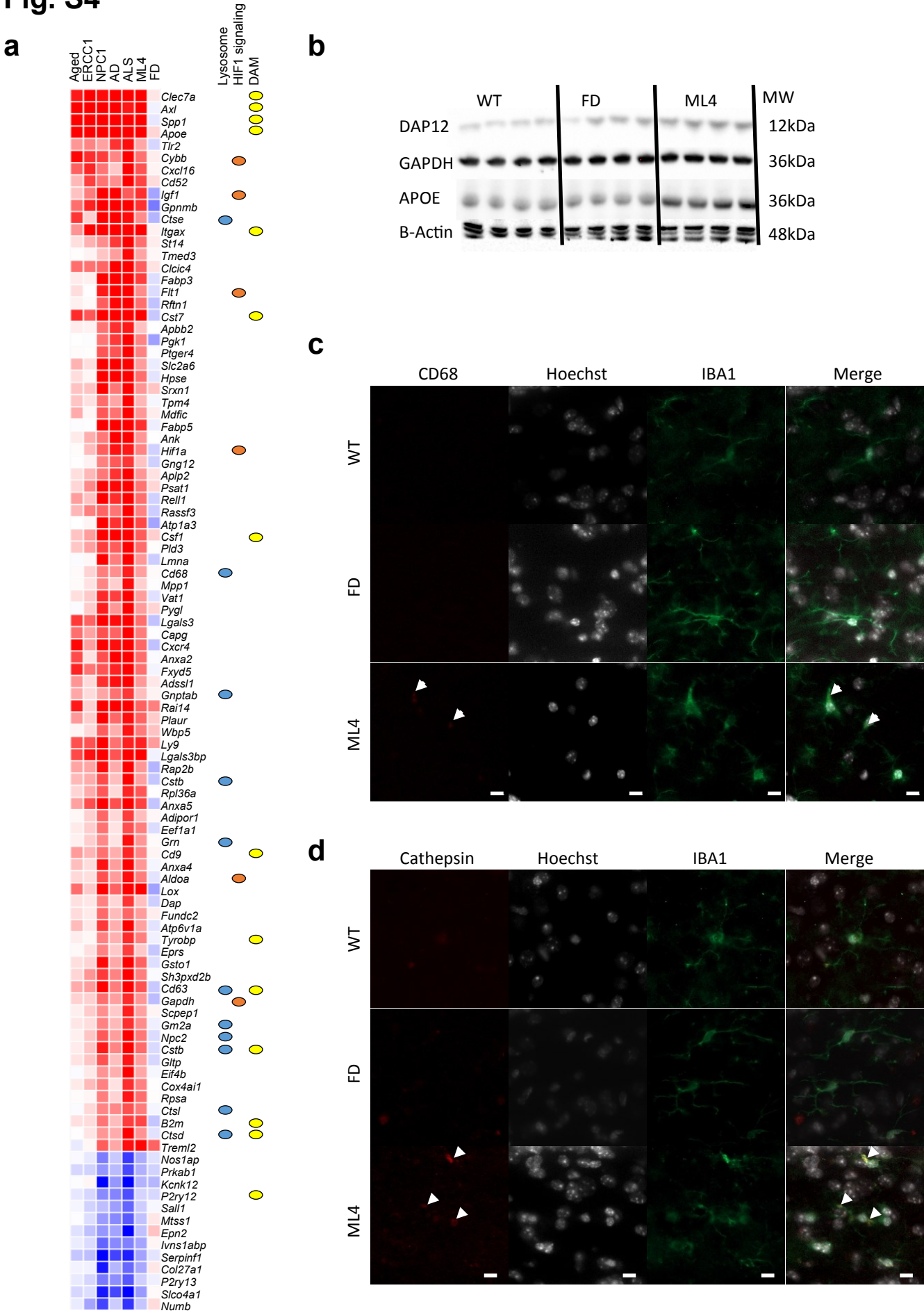

Supplement: Supplementary file 4 — Additional file 4: Figure S4. Expanded Fig. 4c. a Enlarged heatmap with all genes labeled. b Western blot analysis of GAPDH, B-Actin, DAP12, APOE on 100,000 microglia lysates. N=4. c representative IBA1/CD68 immunostaining of WT, FD and ML4 mice hippocampus (DG). d representative IBA1/CTSD immunostaining of WT, FD and ML4 mice hippocampus (DG). Scale bare is 10 μm. [file 12974_2019_1672_MOESM4_ESM.pdf]
