# Supplementary material for: Unique molecular signature in mucolipidosis type IV microglia
Source: J Neuroinflammation. 2019 Dec 28;16:276. doi: 10.1186/s12974-019-1672-4 (PMC6935239; doi:10.1186/s12974-019-1672-4)
Supplement: Supplementary file 2 — Additional file 2: Figure S2. Transcriptomic analysis of 2-month-old Fabry Disease and Mucolipidosis type IV mice microglia. Differential expression was set at + or – 1Log2-fold divergence vs. control with pAdj<0.05. *p<0.01, **p<0.001. Pathway analysis performed with gProfiler (A), ConsensusPathDB (B), STRING (C) and WebGSALT default settings. Data are in Additional file 5: Table S1 and Additional file 6: Table S2. [file 12974_2019_1672_MOESM2_ESM.pdf]

Fig. S2

a

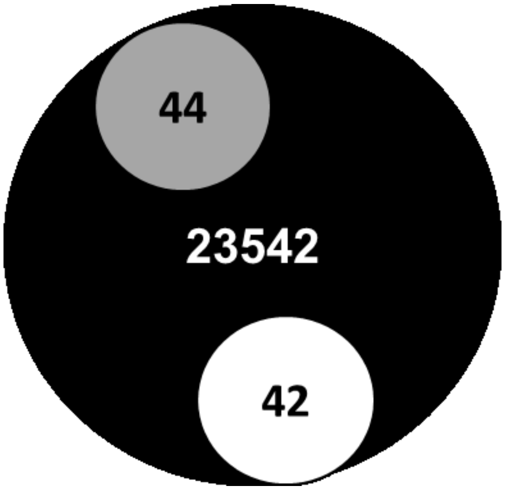

b

Up regulated	A	B	C	D
NOD-like receptor signaling pathway	**	*	***	***
Estrogen signaling pathway	*	0.03		***
mRNA surveillance pathway	0.03		**	**
Prostate cancer	0.03		**	**
T cell receptor signaling pathway	0.04		**	*
B cell receptor signaling pathway	0.05		**	*

c

Down regulated				
Lysosome	0.01		***	**

d

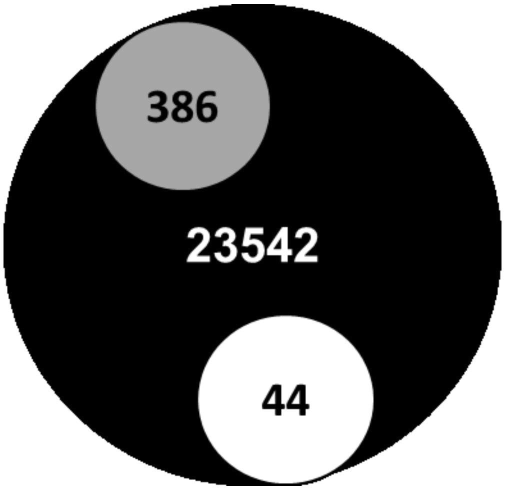

e

Up Regulated	A	B	C	D
Glycosaminoglycan degradation	***	**	***	***
Lysosome	***	***	***	***
Complement and coagulation cascades	***	***	***	***
Parkinson's disease	***	***	***	***
Alzheimer's disease	***	***	***	***
Ribosome	***	***	***	***
Phagosome	***	***	***	***
Rheumatoid arthritis	***	***	***	***
Other glycan degradation	***	***	***	***
Huntington's disease	***	*	***	***
Antigen processing and presentation	***			*
Metabolic pathways	***		***	***

f

Down regulated				
FoxO signaling pathway	*	*		0.01
ErbB signaling pathway	0.03		**	0.03
Fc gamma R-mediated phagocytosis	0.03		**	0.03
Insulin signaling pathway		**	**	0.05
Fc epsilon RI signaling pathway		0.02	**	0.03
